# Life story resources in dementia care: a review

**DOI:** 10.1108/QAOA-02-2014-0003

**Published:** 2014

**Authors:** Jacqueline Kindell, Simon Burrow, Ray Wilkinson, John David Keady

**Affiliations:** Dementia and Ageing Research Team, School of Nursing, Midwifery and Social Work, University of Manchester, Manchester, UK; School of Nursing, Midwifery and Social Work, University of Manchester, Manchester, UK; Department of Human Communication Sciences, University of Sheffield, Sheffield, UK; School of Nursing, Midwifery and Social Work, University of Manchester, Manchester, UK

**Keywords:** Resources, Review, Dementia, Practice, Biography, Life story

## Abstract

**Purpose:**

Life story work has a relatively long tradition in the caring sciences and is recognised as an important component of dementia care and practice. However, to date, there has not been a review of accessible life story resources. The paper aims to discuss these issues.

**Design/methodology/approach:**

Following a systematic approach to identification and inclusion, 11 life story resources were reviewed to ascertain areas of commonality and divergence between the materials.

**Findings:**

The authors were able to group the analysis under eight areas and at the end of this process, it was uncertain if life story work is a formal staff intervention or an informal activity that people with dementia and their families could engage in. Resources also varied in terms of whether the life story information was organised in a chronological way, or with topics of interest/discussion or with a combination of both. Life story evaluation and its impact on the life of the person with dementia is in need of development.

**Practical implications:**

Across the resources the authors identified four reasons to do life story work which the authors have named as: emotional connections; interactional connections; building new connections and practical care connections.

**Social implications:**

There was limited guidance aimed at helping people with dementia to develop and compile their own life story.

**Originality/value:**

This paper provides new insights into the usefulness, future directions and content of life story resources in dementia care. It will be of interest to those in health and social care as well as people living with dementia.

## Introduction

The utility of a personal biography to contextualise and understand ageing has a relatively long history in the gerontological literature and in the “hands-on” care of older people (see e.g. Butler, 1963). Indeed, writing in the mid-1980s, Johnson (1986) states that creating the conditions necessary to help older people tell their story enables a more “dynamic and authentic” (p. 8) picture of their life event(s) to emerge and for helping professions, provides a greater awareness of the person’s present day actions, beliefs and values. Moreover, as all recounted stories stem from individual human experience, Johnson (1986) cautions that such accounts are prone to selective recall and pose a (potentially) distorted reflection of the “truth”.

This is especially evident when stories are recalled through the lens of time, or when past (or present) distressing life events, for example, are cognitively reframed to become more palatable to enable everyday functioning. However, for the helping professions engaged in biographical work, locating the “truth” of the story is not the primary rationale for its undertaking. Rather, what is of importance is for the listener to attempt to understand why the person is sharing that particular story, at that particular time and then attempt to unpack its meaning from within the person’s life course. As Hydén (1997) argues, for older people especially, locating and articulating a biographical connection between past and present life events is one way of preserving the narrator’s personal identity and affirming a sense of agency and self. This connection should not be lightly broken.

For people with dementia, however, the connection between past and present life events can become fractured and the meaning of shared stories difficult to follow. This is because the telling of a chronological life story becomes challenged as a result of cognitive impairment and the sustained, progressive and deleterious impact on the person’s autobiographical and semantic memory functioning, as well as their communication skills. An inability to remember – or misattribute – significant personal life events, such as the names of your children, where you were born and/or the place where you currently live, for example, places the person with dementia at a significant disadvantage when attempting to share their life events and to do so in a sequence that is understood by the listener. This challenge to being understood and validated can place the person with dementia at a serious risk of alienation and stigmatisation by society and perhaps goes some way to explain why people with dementia living in the community experience a shrinking of their social world to one that is largely lived indoors and at home (Duggan et al., 2008). Living with dementia is also to be exposed to loneliness, even within the boundaries of a long-standing relationship (Alzheimer’s Society, 2013).

In his seminal book “*Dementia reconsidered: the person comes first*”, Kitwood (1997) recognised these threats to the personhood of people with dementia and stated that biographical knowledge about a person “becomes essential if that identity is still to be held in place” (p. 56). Kitwood (1997), along with other authors (e.g. Mills, 1998; Keady et al., 2007; Roach et al., 2014), have suggested that one way of holding identity in place is through the conduct, production and use of a life story. In a review of life story work in health and social care by McKeown et al. (2006), life story was seen as an important contribution to caring practice but that the literature was “immature” and that there was a “lack of critical debate about the use of life story work in practice” (p. 241). In dementia care, life story work has gained a foothold in practice (Haight et al., 2003; Keady et al., 2005; Kellett et al., 2010) and this has led to a number of life story resources being produced that are aimed at helping practitioners (and families) link the person with dementia’s past to the present. Whilst limited in their number, a range of objectives and positive outcomes for the person with dementia, their families and staff in conducting life story work, have also been described in the literature; for example, a recurring theme appears to be the importance of “connections” including developing relationships in care (Kellett et al., 2010) and fostering communication (McKeown et al., 2006). We will return to the theme of connections at the end of the paper.

However, what has not been attempted before, is a review of the available and accessible life story resources in dementia care and what may be the core and divergent contents of such material. This paper attempts to fill this current gap in the knowledge base and provide pointers for the future development and application of such resources.

## Review methods

The primary aim of the review is to systematically document the available and accessible life story resources in dementia care in the UK. We based our definition of life story work on the contribution of Murphy (1994) who viewed life story work as a process carried out on a one-to-one basis for personal reasons, rather than as an oral history sharing exercise, or as a reminiscence activity aimed at locating shared experiences. To do this we targeted practical written resources in the form of “life story templates/booklets” or “how-to-guides” that could either be bought from a publisher and/or were available for download on the internet. We excluded articles in practice journals and chapters in publications that offered advice/tips on life story work, materials not aimed at older people or people with dementia. To perform the search, two of the authors (JK and SB), undertook separate searches using recognised electronic data bases (Medline, PsycINFO, ERIC and PubMed) using the key words “life story work” in combination with the MeSH terms: life story review; dementia; Alzheimer’s; biography; biographical; dementia; resources; materials.

A web-based search of materials using the Google search engine was also performed. The two authors then came back together in a follow-up meeting to compare and discuss their respective search findings. At the end of this meeting, a final, agreed list of 11 life story resources was identified that matched the inclusion criteria.

The life story resources that were available for purchase were bought and the contents of all resources were read and thematically analysed (Braun and Clarke, 2006). This process of analysis was conducted in three phases: first, independently by three of the authors (JK, SB and JDK); second, in a different combination of pairs to agree emergent themes (JK-JDK; JK-SB; JDK-SB); third, as a group of three to pool analysis and reach consensus on the core/divergent areas of the resource contents. A framework for mapping the contents of each of the life story resources was then devised by the authors and the results of this exercise are shown in Table I. Table I will be referred to in the next section and we will use the allocated number in the Table – using square brackets – to identify the resource, e.g. Told in South Yorkshire [11].

## Findings

### Presentation of resources

There was a great variety of resources available, including downloadable sheets, booklets to complete, books and boxed multimedia resources. These ranged from free resources on the web from organisations sharing their practice (My Life Story [2], Life Story Template [4], Told in South Yorkshire [11]) through to more expensive resources from specialist publishing companies (Portrait of a Life [8] at £422.50 was the most expensive). Some resources consisted of detailed guidance on life story books and work, whereas others consisted of templates of varying lengths to complete. The resources dated as far back as 1993. It was notable that an early well-regarded resource (It Started with a Sea-Shell [5]) remains relevant 20 years later, for example, in the advice to start life story work with the person with dementia and that, by doing so, created a resource “that reflects the issues and events of most importance to that person” (p. 16).

### Rationale and purpose

Examining these resources indicates that life story work has the potential to be a diverse activity, carried out in a variety of settings, in different ways, using different materials, by a variety of people, with potentially different objectives. The resources, for example, varied in terms of the stated reasons for doing life story work. In some resources there is extensive definition of life story work (e.g. It Started with a Sea-Shell [5] and Portrait of a Life [8]) and in others, there was little or no formal definition, e.g. My Life Story [7] and Activities to Share [6]). Where formal definitions were provided, these seemed to emphasise the therapeutic value of life story work. In some instances life story work was presented as part of a broader activity, for instance in gathering relevant information to write a care plan (Enriched Care Planning [3]) or providing activities that were more personally relevant (All About Me [1]). In other instances, life story work was seen as a valuable “stand-alone” activity in its own right with a view to gathering and organising facts and memories into a personalised autobiography (My Life Story [7]) or, indeed, with a primary aim to “have fun” (Remembering Together [9]).

### Who is the resource aimed at?

Some resources were aimed at older people in general (e.g. All About Me [1], Getting to Know Me [10]), whereas others were particularly focused on dementia (e.g. Enriched Care Planning [3], It Started with a Sea-Shell [5], Portrait of a Life [8]). Some resources were clearly viewed by the authors as an activity for an older person to do either on their own or with a relative or caregiver: My Life Story [7], Remembering Together [9], Getting to Know Me [10]. Other resources, however, were clearly aimed by the authors at a professional audience stressing the therapeutic value of life story work for health and social care staff to carry out, or facilitate, with those in their care. Portrait of a Life [8] for example had a written consent form for the service user and carer to sign in order to “consent for involvement in life story work”, clearly indicating the clinical nature of the process in this case. In contrast, Told in South Yorkshire [11] stated that consent sheets were not necessary. Resources aimed at staff sometimes included information relating to care practices such as help with dressing, as seen in My Life Story [2] or food and nutrition in Portrait of a Life [8].

### Accompanying guidance

Resources varied hugely in their accompanying guidance, ranging from none, to a page or so, on to extensive multimedia advice. Those with more extensive advice were often aimed at a professional audience (e.g. Portrait of a Life [8], Told in South Yorkshire [11]) and therefore considered the challenges and benefits of delivering life story work in health and social care settings as a therapeutic intervention or activity to enhance care.

### Flexibility and creativity in product and process

Many of the resources with accompanying guidance stressed the need to be flexible and creative when engaging in life story work. This included the need to be sensitive when talking to the person as traumatic and upsetting past memories could be brought to the fore. Whilst a number of the resources mentioned alternative formats to life story books, or information sheets, and some gave innovative suggestions of what these might be (e.g. Told in South Yorkshire [11]), it was only Portrait of a Life [8] and the Life Story Template [4] that gave any practical advice about how to go about this, giving advice on how to make a memory box or story board.

### Template use and format

There was a variety of ways the “template” or advice was to be used. In some instances the template was to be written – or typed directly into – with photographs added (Life Story Template [4], My Life Story (Activities to Share) [6]), whereas in others the template was a means to an end through either producing other life story resources such as a book or memory box or contributing to a personalised care plan or activity programme. Resources varied in terms of whether the information was organised in a chronological way, or with topics of interest/discussion or with some of each. This appears, in part, to reflect differences in focus between writing one’s life story, as a chronological process, or an activity to get to know the person better. Particularly with the older templates, there were instances of gender stereotyping and instances of templates not taking account differences in sensory needs, life style, culture and sexuality.

### How the resource is to be used

Even in the most extensive resources there was only limited guidance on how to apply the life story or how to use the resource developed in the longer term. Perhaps the exception to this was Enriched Care Planning for People with Dementia [3] where there was clear guidance on how to incorporate the information into an on-going care plan and to review this. As a consequence, there was generally more focus on the collection of information and the production of a life story resource, rather than on how that information might be used to benefit the person with dementia once the initial life story process has been completed.

### Evaluation of the resource

There appeared to be no standard way of assessing the outcomes of life story work, although many positive outcomes were stated including: more person centred activities, care plans and conversation. At a superficial level it was relatively easy to “measure” if a template had been completed or a resource (e.g. a life story book) had been made, but at a deeper more sophisticated level, in none of the resources were there any ways suggested to systematically evaluate if such resources were used or enjoyed by the person with dementia and their carers on an on-going basis. Where evaluations were encouraged, these were often descriptive in nature or used measures that might not be sensitive to, or appropriate for, the outcomes expected from life story work such as Folstein *et al*.’s (1975) Mini-mental State Examination (Portrait of a Life [8]).

## Discussion

As this review has demonstrated, there are a range of resources available to facilitate life story work with people with dementia. Taken as a whole, however, there were mixed messages as to whether life story work was a formal intervention for staff to “control” and “facilitate”, or an informal activity that people with dementia and their families could engage in without recourse to formal help, training or materials. In some quarters, life story appears to be becoming a professionalised intervention, rather than an informal activity, and perhaps the term “Life Story Work” is therefore fitting for this purpose. Those resources aimed at a professional audience were more likely to consider the complexities of carrying out life story work in clinical practice (e.g. It Started with a Sea-Shell [5], Portrait of a Life [8]) consistent with many themes raised in the literature (McKeown et al., 2013b; Scherrer et al., 2014).

In considering the benefits of life story work in dementia, the focus appears to be within the mid to later stages of the condition, often when services are more heavily involved, rather than in the early stages. There is limited guidance for people with dementia to develop their own life story and have control over what they wish to share now and for the future. Interestingly, most of the resources with advice stress that life story work is only the starting point of a bigger process and that creativity and flexibility are required. However, one has to ask if the nature of the template facilitates or constrains creativity, or if creativity, in reality, lies in the hands of the person(s) completing the life story. As with many things in dementia care, sensitive and flexible communication skills are often required and Told in South Yorkshire [11] outlines the qualities needed by the person gathering the life story.

In Getting to Know Me [10], the author stresses the need to remember that “this is not an official document completed for bureaucratic purposes, it is a personal account done purely for personal purposes” (p. 7). Reinforcing the personal nature of this work seems excellent advice; however, even the most creative of life story resources and formats has the potential to be reduced to a rigid clinical encounter if the individual delivering the activity feels they must fill in each box in a prescribed order, have photographs for every life event or have a life story book for every service user. Indeed, cognitive tests are available that test autobiographical memory (e.g. Kopelman et al., 1990) that have very similar formats to those life story resources with chronological templates. Arguably, the difference between performing such a clinical test and facilitating life story work with an individual with dementia may lie solely in the communication skills of the worker.

There also remains the issue of the chronological nature of some of the resources. As discussed, the fragmented nature of communication and memory in dementia may make it difficult for many people to recall and recount events in such an ordered fashion and therefore compromise their ability to play a part in the process. Instead, a more flexible and naturalistic encounter might allow the worker to follow the lead of the person with dementia in conversation and, in the process, find out those things that are particularly important to the individual concerned.

The issue of upsetting memories, or the revelation of private stories, is often raised in the resources. This is likely to be a particular problem for staff getting to know a person, as they are unaware of memories likely to cause upset. A spouse, in contrast, may already be armed with this knowledge, though their shared life history with the person with dementia and therefore may be able to use this knowledge to facilitate or decode communication, in ways that a staff member is not yet able to do. Within the reviewed resources, there seems to be general agreement that such issues should not be placed in a life story resource. However, for staff, this raises the question of whether, and where, to record such information. Decisions around this issue are likely to take into account whether this knowledge influences behaviour or care in any way, now and in the future, as consideration of life story is an important aspect to assessment of behaviours that challenge, for example (as an illustration see: James, 2011).

Across these resources and, arguably, in clinical practice, life story books dominate as the preferred format, in some instances requiring time and a fair degree of technical and computing skills in scanning, moving images and adding text. Alternative formats such as memory boxes, films, sounds are sometimes being used and these, therefore, go beyond the visual and textual format found in life story books and exploit other senses, as has often been advocated in reminiscence work (Schweitzer and Bruce, 2008). It also appears that life story work could be seen as an activity to spark off conversation about the past, in a remote way, rather than as an opportunity to find out about activities that can then be directly experienced. In this way there was often a focus on talking about one’s experiences (e.g. liking cars), rather than directly (re−) experiencing them (e.g. going out for a ride or rummaging through old car parts in a real garage). Life story work may therefore lack a multisensory and embodied dimension that requires further exploration in research and practice.

Finally, there remains the thorny issue of evaluation of life story work. This, in many respects, stems from the diversity of life story work itself and indeed if it is a clinical intervention or not. As mentioned a little earlier, whilst it is relatively easy to measure if a life story book has been completed with a given individual, it is less easy to measure the impact of this book on their lives. In research and clinical practice outcomes such as enjoyment and “having fun” (Remembering Together [9]), rarely impress managers and grant funders. However, for a person with dementia and their carer, this may be their primary goal. In practice and research there may be many different and potentially multiple outcomes from life story work and these stem from differences in initial goals for the work with a given individual.

## Recommendations for practice and research

Our primary recommendation for both practice and research is the need to clarify the focus and goals for life story work with the person with dementia. This focus enables appropriate outcomes to be defined and explored (see also: McKeown et al., 2013b). Across the resources there seemed to be a variety of reasons to do life story work and potential for different disciplines to focus on different objectives. Whilst “making connections” was a recurrent theme in the resources, there were potentially different points of connection for this work and we felt the following formulation may help clinicians and researchers explore this further in their work:

■ Emotional connections: as a psychological process with couples/families to help them positively connect as a couple or family unit, or help them get through a difficult time (e.g. post diagnosis; increasing or changing a care plan). To help the person with dementia connect with their own identity in a positive way and to feel valued and feel that they matter. In this area the focus and outcome is emotions and coping at a psychological level.
■ Interactional connections: to produce a resource (book, board, box) that in an on-going way can support memories and conversation around personally related topics and through increased shared knowledge can help to decode potentially “empty speech”. The outcome of this might be visible in the analysis of interactions that are “in the moment” and captured in visual media, such as through a series of photographs that reveal joyful and happy facial expressions of the person with dementia.
■ Building new connections: as a process to help build supportive relationships and partnerships in care between staff, the person with dementia and their relatives. This includes providing an overt challenge to depersonalised care or practice. An important aspect to this may be analysis of attitudes and satisfaction with care.
■ Practical care connections: in order to provide an appropriate care plan and activities for a person with dementia built around their particular needs. An outcome of this might be a care plan that contains specific autobiographical information that is recognisable as pertinent to only a certain individual at audit, should the life story work be conducted in a formal care setting.

By defining individual therapeutic goals this would allow their measurement to be individualised from within the person’s relational and lived experience.

Our other recommendation lies in the need to develop more explicit guidance in life story work that is flexible and begins with the stories the individual with dementia wants to share, rather than the questions the worker wishes to ask. We suspect this approach already exists in the practice of more experienced workers, however, systematically unpicking this tacit expertise would enhance the field considerably.

Jacqueline Kindell, Simon Burrow, Ray Wilkinson, John David Keady. Published by Emerald Group Publishing Limited. This paper is published under the Creative Commons Attribution (CC BY 3.0) licence. Anyone may reproduce, distribute, translate and create derivative works of this paper (for both commercial and non-commercial purposes), subject to full attribution to the original publication and authors. The full terms of this licence may be seen at http://creativecommons.org/licences/by/3.0/legalcode

## Figures and Tables

**Table 1:** Resources reviewed

*Publisher and presentation*	*Rationale/purpose of resource, including definition of life story work*	*Guidance: for production and use of resource?*	*Is there a template? Is process chronological?*	*Who aimed at? Who has control over process?*	*Final product & evaluation of resource/work?*
(1) *All about me* National Association for Providers of Activities for Older People(NAPA), a UK Charity. Template consisting of a 12 page bound booklet on glossy paper with questions and prompts and you can write directly into the booklet. Cost £2.40 from web site: http://napashop.mamutweb.com/Shop	No definition, not setting out to do life story work as a stand-alone process. Aims to gather information about a person’s life story and current lifestyle choices – as the starting point for improved activity provision Not aiming to produce a book or look through template with person	No guidance	A template: chronological then moves into various topics	Staff – by implication the guidance is generally aimed at staff carrying out activities with older people, particularly in care settings	Information about the person’s life that can aid activity planning. No discussion of this or evaluation
(2) *My Life Story* Dementia UK and the Central and North West London NHS Foundation Trust. A template (A4 word document) for life story work that can be written or typed into. Free from: www.dementiauk.org/information-support/life-story-work/	A definition of life story work is on the web site and within the guidance The template is a starting point to help professionals gather information to deliver person centred care and to develop a life story resource, e.g. book, DVD, box, etc	Additional guidance to download that covers the process and making the product. No guidance on using the resources developed	A template: essentially chronological	Staff providing health and social care	A life story resource A report & article describe evaluation of a life story project where the template was used. Includes 2 questionnaires for staff & carer/patients on the value of life story work
(3) *Enriched Care Planning for People with Dementia* May *et al*. (2009), Jessica Kingsley Publishers London. A 174 page book that includes a life story profiling template	Chapter “Life Story” discusses the approach including the benefits. Aims to provide a more personalised and enriched care plan in order to deliver person centred care. Thus the information gathered feeds into this process in a systematic. The outcome would be a better care plan	Extensive guidance within Life Story chapter and others on development and review of enriched care plan that takes account of life story	A template: chronological and advice is to start with early memories	Staff providing health and social care	Information about the person’s life to aid development of an enriched care plan. Discusses ways to set goals & review care plan but not specifically life story work
(4) *Life Stories* Dementia Care, a UK Charity A4, A5 or A6 life story templates that you can download consisting of prompt questions and spaces to enter written information and photographs. Also information on how to make a memory box or one page summary. Free from: www.dementiacare.org.uk/living-well-with-dementia/i-am-a-carer-or-friend/planning-for-today-or-tomorrow/life-stories	Some discussion of importance of life story and what a life story book might be, with an example provided Aim is to make a life story book/resource/memory box. The templates are designed to “get you started”	Some guidance notes on books and memory boxes and putting life story work into action. Sample life story book on the web site for illustration	A template: starts with people and likes/dislikes and moves on to chronology	Aimed at carers (writing with the person with dementia they care for) and the person writing their own life story, as well as staff	Life story resource. No discussion of evaluation
(5) *It Started with a Sea-Shell: Life Story Work and People with Dementia* Murphy (1994), Dementia Services Development Centre, University of Stirling. A 30 page booklet of guidance with a “personal history template” for relatives to complete in appendix. Free from: www.dementiashop.co.uk/	Extensive definition of life story work, including how it differs from life review and reminiscence. Detailed description of why do it and the potential outcomes for person with dementia, carers and staff	Extensive guidance and discussion about how to deliver life story work & how to get it started in the workplace as an integral part of assessment and care	Guidance along with a relative’s template: template is chronological but advice is not necessarily to deliver life story work this way	Staff providing health and social care. Acknowledges need to develop methods to get carers to do work in community	Life story resource. Suggests potential outcomes for life story work but not any way to assess benefit at an individual level
(6) *My Life Story* Activities to Share. A 32 page bound life story booklet consisting of prompt questions and spaces to enter information by writing on the lines and adding photos. £4.50 if bought individually: www.activitiestoshare.co.uk/p/125/life-story-books	No formal definition of life story work, but discusses life story books. Aims to deliver more person centred care, develop connections and relationships between staff and service users, improved sense of identity and self-worth in the person. Also process itself described as an enjoyable activity	Guidance notes on the back cover	A template: chronological and then topics	Staff and volunteers	A book containing information about the person’s life. No discussion about evaluation
(7) *My Life Story* Produced by Springfield Leisure-Art Collection (1993). A 124 page spiral bound life story book consisting of prompt questions and spaces to enter written information and photographs. Available from Winslow Press, £33.95: www.winslowresources.com/my-life-story.html	Does not give a definition of life story work. Resource aims to organise facts and memories into a personalised autobiography. Not a dementia specific resource, could be used by anyone attempting to write a life story	Guidance notes within book, but about how to facilitate information or clarify questions. No info on on-going use	A template: largely chronological	Author states “anyone”. But publisher aims this at “the professional” on the web site	A book containing extensive information about a person’s life. No discussion of evaluation
(8) *Portrait of a Life: a Multimedia Toolkit for Life Story Work* Wightmanef al. (2009)., South West Yorkshire Partnership NHS Foundation Trust. A plastic folder containing: A 97 page written guide, 2 DVD’s and a CD-Rom. Cost: £345 = VAT from www.medipex.co.uk/success-stories/portrait-of-a-life/ Now available as an e-learning resource	Provides a definition and rationale for life story work. Toolkit aims to be “a one-stop-shop to help in the undertaking of life story work” and therefore the purpose and benefits of life story work are laid out in detail	Extensive guidance in written manual and on DVD’s. Contains advice on making books, boxes and story boards and challenges of undertaking life story work	Guidance along with a template: “The CARER model life history” is not chronological	The resource states toolkit can be used by individuals and their family carers, but cost & format indicate a staff coordinated process. Carers complete information template with person with dementia where possible	A life story resource. Contains an evaluation sheet and a reflective practice form. Evaluation sheet has boxes to describe outcomes, and MMSE and HADS as baseline assessments that could be repeated
(9) *Remembering together: Making aLife History Book* Alzheimer’s Society, UK Charity. A 4 page guidance leaflet Free from: www.alzheimers.org.uk/site/scripts/ documents_info.php?documentlD = 1671	Gives a definition of a life story book. Main emphasis is to “have fun” and is on the activity of making the book, although final product is mentioned	Guidance provided on how to make the book and stresses involvement of person	No template provided, this is guidance. Advises does not need to be chronological	Carer focused but does discuss the person with dementia writing their own story themselves if able	A life story book. No discussion of evaluation
(10) *Getting to Know Me* Midwinter (1996). A 24 page A5 bound booklet that you write into. Available from Winslow press for £3.95: www.winslowresources.com/my-life-story.html	No definition of life story work. A checklist to help get to know the person, to develop a portrait of their life and develop a more rounded view of them in order to “grasp the wholeness of the older person” Not a dementia specific resource, could be used by anyone attempting to write a life story	Guidance provided on how to use resource	A template: in part chronological with other topics and information	Anyone in day to day contact with older people. Can be completed by carer or person themselves	A book containing details of the person’s life. No discussion about evaluation
(11) *Told in South Yorkshire-Life Story Work & People with Dementia* McKeown *et al*. (2013a). A 19 page guidance document with further information on web site. Free from: www.toldinsouthyorkshire.co.uk/blog/	Definition of life story work provided along with an outline of the benefits and challenges. Aimed at those working with people with dementia. Contains information to get people started in making a variety of resources	Guidance provided on different ways to undertake life story work. Some advice on other formats other than books	No template provided, this is guidance. Encouraged to start with what the person wants to tell you rather than chronological	Staff led process. Putting person at centre stressed	A life story resource. Suggests potential outcomes but no systematic way to assess these
